# Alzheimer’s disease and epilepsy: The top 100 cited papers

**DOI:** 10.3389/fnagi.2022.926982

**Published:** 2022-07-22

**Authors:** Gui-Fen Zhang, Wen-Xin Gong, Zheng-Yan-Ran Xu, Yi Guo

**Affiliations:** ^1^Department of General Practice and International Medicine, Second Affiliated Hospital, School of Medicine, Zhejiang University, Hangzhou, China; ^2^Department of Neurology, Epilepsy Center, Second Affiliated Hospital, School of Medicine, Zhejiang University, Hangzhou, China

**Keywords:** Alzheimer’s disease, epilepsy, bibliometric study, top-cited, citation

## Abstract

**Background:**

Alzheimer’s disease (AD) is one of the common neurodegenerative diseases, which often coexists with epilepsy. It is very significant to study the treatment options and the relationship between AD and epilepsy.

**Aims:**

The purpose of this study was to analyze the top 100 cited papers about AD and epilepsy using bibliometrics, and to describe the current situation and predict research hot spots.

**Methods:**

Top 100 papers were obtained from the Web of Science Core Collection (WoSCC). The WoSCC was used to analyze the author, institution, country, title, keywords, abstract, citation, subject category, publication year, impact factor (IF), and other functions. SPSS25 software was used for statistical analysis and CiteSpace V.5.7.R2 was used to visualize the information through collaborative networks.

**Results:**

The number of publications gradually increased from 2000 to 2021. The total citation count for the top 100 papers ranged from 15 to 433(mean = 67.43). The largest number of papers were published in 2016 (*n* = 11). Meanwhile, USA (centrality: 0.93) and Columbia University (centrality: 0.06) were the most influential research country and institutions, respectively. The top contributing journals was Journal of Alzheimer’s Disease (8%). The IF for journals ranged from 1.819 to 53.44. A network analysis of the author’s keywords showed that “beta” (centrality: 0.39), “amyloid beta” (centrality: 0.29), “hyperexcitability” (centrality: 0.29) and “disease” (centrality: 0.29) had a high degree of centrality.

**Conclusion:**

AD and epilepsy have been intensively studied in the past few years. The relationships, mechanisms and treatment of AD and epilepsy will be subjects of active research hotpots in future. These findings provide valuable information for clinicians and scientists to identify new perspectives with potential collaborators and cooperative countries.

## Introduction

Alzheimer’s disease (AD) and epilepsy are common neurological diseases. The number of patients with dementia and epilepsy in the global population is increasing, representing a growing problem for global health. The overall lifetime prevalence of epilepsy is 7.60 per 1,000 population [95% confidence interval (CI) 6.17–9.38] and the prevalence of epilepsy tends to peak in the elderly (Beghi, 2020). The prevalence of AD in Europe was estimated at 5.05% (95% CI, 4.73–5.39), and similar to epilepsy, AD prevalence increases with age (Niu et al., 2017). In fact, these two diseases are related. Epilepsy occurs more frequently in patients with AD than in those with non-Alzheimer’s disease (Samson et al., 1996). Recent findings showed that seizures could accelerate the decline of cognitive ability in patients with AD and that there might be an important bidirectional relationship between epilepsy and AD (Sen et al., 2018). AD and Epilepsy also share many pathological similarities (Lehmann et al., 2021), e.g., temporal lobe atrophy, neuronal death, gliosis, neuritic alterations, and neuroinflammation (Struble et al., 2010). In addition, AD plus epilepsy would lead to more serious clinical consequences, such as cognitive decline, weakness, anxiety, depression, social withdrawal, psychological and behavioral comorbidity, and poor treatment compliance (Cretin, 2021). Overtime, a large amount of literature has been published comprising a wide range of relevant research and clinical themes. However, the precise mechanisms leading to the development of seizures in the setting of AD are still under investigation and require further study. A meta-analysis showed that the quality of evidence on the treatment outcome of epilepsy in patients with AD was very low (Liu and Wang, 2021).

As a discipline emerging since its formal foundation, a bibliometric review of the literature was warranted to aid the synthesis and implementation of the evidence base. Despite citation analysis across a broad range of neurosciences (Yeung et al., 2017), there is limited information in the field of AD and epilepsy, with few published studies. Citation counting is an important metric to understand the significance of the contribution of research to a research field (Fox et al., 2021). Previous reviews only relied on individuals to study the research through literature summary and extraction, and thus cannot fully reflect the temporal and spatial distribution of researchers, institutions, and journals. Moreover, it is difficult to visualize the internal structure of the knowledge base and research focus, and systematic, comprehensive, and visual research are rarely found. Therefore, the present study aims to comprehensively analyze the current status, research hotpots, and development trends through a bibliometric analysis of the top 100 papers on AD and epilepsy published from 2000 to 2021. The findings may help follow-up researchers study the association between AD and epilepsy, identify journal publications and collaborators, and analyze keywords and research trends, which might promote research aiming to determine the cause, mechanism, and treatment of the disease.

## Materials and methods

### Data source

**FIGURE 1:**
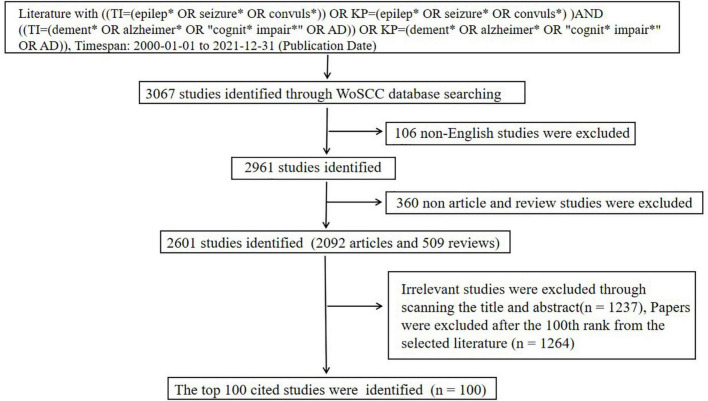
Flow chart of literature collection.

The retrieval data for measurement and statistical analysis were screened from the Web of Science Core Collection (WoSCC), which provided the citation search, giving access to multiple databases that reference cross-disciplinary research and allowing an in-depth exploration of specialized subfields (Wu et al., 2021). We conducted a literature search from the WoSCC on June 5th, 2022. In this study, the search criteria in the WoSCC database were as follows: ((TI = (epilep* OR seizure* OR convuls*)) OR KP = (epilep* OR seizure* OR convuls*)) AND ((TI = (dement* OR Alzheimer* OR “cognit* impair*” OR AD)) OR KP = (dement* OR Alzheimer* OR “cognit* impair*” OR AD)). Timespan: 2000-01-01 to 2021-12-31 (Publication Date). Document type: articles and reviews; Language: English. A total of 2601 records were retrieved. Then, two independent investigators reviewed the titles and abstracts and deleted studies that were not associated with AD and epilepsy, which excluded 1237 papers according to the criteria (Guo et al., 2021). And 1264 papers were excluded after the 100th rank from the selected literature. Finally, the top 100 studies were determined. A Preferred Reporting Items for Systematic Reviews and Meta-Analyses (PRISMA) flow diagram for the WoSCC results is provided in Figure 1.

### Data analysis

In the present study, WoSCC was used to analyze the author, institution, country, title, keywords, abstract, citation, subject category, publication year, impact factor (IF), and other functions. The selected documents were imported into Excel (Microsoft Corporation, Redmond, WA, United States) and CiteSpace 5.8.R3 (64-bit) (Palop and Mucke, 2009). SPSS 25.0 statistical software (IBM Corp., Armonk, NY, United States) was used for statistical analysis. Continuous variables were expressed as the mean ± SD. Categorical variables were expressed as a percentage. CiteSpace, a bibliometric analysis tool, was created by Dr. Chaomei Chen (School of Information Science and Technology, Drexel University, Philadelphia, PA, United States) and his team. It visualizes countries/regions, institutions, authors and their cooperative relationships, co-cited references, and co-occurrence words through collaborative networks, which has been widely used in biomedical research fields.

Three folders of the research were created, including input folder placing the data downloaded from WoSCC, a data folder containing the data after deleting duplicate documents, and a project folder containing the data processed by cite. We did not find duplicate documents that needed to be deleted. The overall selected time span was from January 2000 to December 2021. Then, the slice length was set as 2 years. The node type was selected according to the type of analysis performed. The link lines between the nodes indicated the collaborative relationships. The size of the circles represented the number of papers published by the country/region, institute, or author. Purple rings indicated that these countries/regions, institutes, or authors had greater centrality.

## Results

### The 100 most-cited publications

We retrieved the 100 most frequently cited papers related to AD and epilepsy. The results were ranked according to citation counts to represent the 100 most-cited publications (76 articles and 24 reviews). A comprehensive list of the 100 publications and a citation details are presented in the Table 1.

**TABLE 1:** The 100 most-cited publications.

Rank	Article title	Journal	IF (2020)	Times Cited	Times Cited, in 2021	Times Cited, per year
1	Epilepsy and Cognitive Impairments in Alzheimer Disease	ARCHIVES OF NEUROLOGY	7.419	433	39	31
2	Amyloid beta-Induced Neuronal Hyperexcitability Triggers Progressive Epilepsy	JOURNAL OF NEUROSCIENCE	6.167	405	54	28.93
3	Incidence and predictors of seizures in patients with Alzheimer’s disease	EPILEPSIA	5.886	391	37	23
4	Seizures and Epileptiform Activity in the Early Stages of Alzheimer’s Disease	JAMA NEUROLOGY	18.302	357	56	35.7
5	Epileptic activity in Alzheimer’s disease: causes and clinical relevance	LANCET NEUROLOGY	44.182	213	64	35.5
6	Incidence and Impact of Subclinical Epileptiform Activity in Alzheimer’s Disease	ANNALS OF NEUROLOGY	10.442	201	43	28.71
7	Antisense Reduction of Tau in Adult Mice Protects against Seizures	JOURNAL OF THE INTERNATIONAL NEUROPSYCHOLOGICAL SOCIETY	6.167	192	31	19.2
8	Seizures in Alzheimer Disease Who, When, and How Common?	ARCHIVES OF NEUROLOGY	7.419	187	21	13.36
9	Silent hippocampal seizures and spikes identified by foramen ovale electrodes in Alzheimer’s disease	NATURE MEDICINE	53.44	172	54	28.67
10	A perfect storm: Converging paths of epilepsy and Alzheimer’s dementia intersect in the hippocampal formation	EPILEPSIA	5.886	167	15	13.92
11	Tau Loss Attenuates Neuronal Network Hyperexcitability in Mouse and Drosophila Genetic Models of Epilepsy	JOURNAL OF NEUROSCIENCE	6.167	148	26	14.8
12	Seizures and Epilepsy in Alzheimer’s Disease	CNS NEUROSCIENCE AND THERAPEUTICS	5.243	135	23	12.27
13	Seizures in elderly patients with dementia - Epidemiology and management	DRUGS AND AGING	3.923	132	8	6.6
14	Down syndrome, Alzheimer’s disease, and seizures	BRAIN AND DEVELOPMENT	1.961	128	8	7.11
15	Hyperphosphorylated tau in patients with refractory epilepsy correlates with cognitive decline: a study of temporal lobe resections	BRAIN	13.501	123	28	17.57
16	Levetiracetam, lamotrigine, and phenobarbital in patients with epileptic seizures and Alzheimer’s disease	EPILEPSY AND BEHAVIOR	2.937	113	23	8.69
17	Septal networks: relevance to theta rhythm, epilepsy and Alzheimer’s disease	JOURNAL OF NEUROCHEMISTRY	5.372	105	9	6.18
18	Prevalence and causes of seizures at the time of diagnosis of probable Alzheimer’s disease	DEMENTIA AND GERIATRIC COGNITIVE DISORDERS	2.959	94	8	5.53
19	Cognition and dementia in older patients with epilepsy	BRAIN	7.419	93	31	18.6
20	Seizures in patients with Alzheimer’s disease or vascular dementia: A population-based nested casecontrol analysis	EPILEPSIA	5.886	89	14	8.9
21	Incidence of New-Onset Seizures in Mild to Moderate Alzheimer Disease	ARCHIVES OF NEUROLOGY	7.419	82	11	7.45
22	Seizures in Alzheimer’s disease	NEUROSCIENCE	3.59	81	13	10.13
23	Shared cognitive and behavioral impairments in epilepsy and Alzheimer’s disease and potential underlying mechanisms	EPILEPSY AND BEHAVIOR	2.937	80	9	8
24	Epileptic Seizures in AD Patients	NEUROMOLECULAR MEDICINE	3.843	78	3	6
25	Seizures in Alzheimer’s Disease: Clinical and Epidemiological Data	EPILEPSY CURRENTS	7.5	71	11	6.45
26	Spontaneous epileptiform discharges in a mouse model of Alzheimer’s disease are suppressed by antiepileptic drugs that block sodium channels	EPILEPSY RESEARCH	3.045	65	9	5.42
27	Relative Incidence of Seizures and Myoclonus in Alzheimer’s Disease, Dementia with Lewy Bodies, and Frontotemporal Dementia	JOURNAL OF ALZHEIMER’S DISEASE	4.472	59	22	9.83
28	Epileptic Seizures in Alzheimer Disease A Review	ALZHEIMER DISEASE AND ASSOCIATED DISORDERS	2.703	59	11	8.43
29	Interictal spikes during sleep are an early defect in the Tg2576 mouse model of beta-amyloid neuropathology	SCIENTIFIC REPORTS	4.38	59	11	8.43
30	Epileptic Prodromal Alzheimer’s Disease, a Retrospective Study of 13 New Cases: Expanding the Spectrum of Alzheimer’s Disease to an Epileptic Variant?	JOURNAL OF ALZHEIMER’S DISEASE	4.472	58	13	8.29
31	Levetiracetam monotherapy in Alzheimer patients with late-onset seizures: a prospective observational study	EUROPEAN JOURNAL OF NEUROLOGY	6.089	58	8	3.63
32	Common factors among Alzheimer’s disease, Parkinson’s disease, and epilepsy: Possible role of the noradrenergic nervous system	EPILEPSIA	5.886	57	3	5.18
33	Dietary energy substrates reverse early neuronal hyperactivity in a mouse model of Alzheimer’s disease	JOURNAL OF NEUROCHEMISTRY	5.372	56	5	5.6
34	Brivaracetam, but not ethosuximide, reverses memory impairments in an Alzheimer’s disease mouse model	ALZHEIMER’S RESEARCH AND THERAPY	6.982	54	12	6.75
35	Increased Cortical and Thalamic Excitability in Freely Moving APPswe/PS1dE9 Mice Modeling Epileptic Activity Associated with Alzheimer’s Disease	CEREBRAL CORTEX	5.357	51	8	5.1
36	Down Syndrome and Dementia: Seizures and Cognitive Decline	JOURNAL OF ALZHEIMER’S DISEASE	4.472	51	8	4.64
37	The efficacy of a voxel-based morphometry on the analysis of imaging in schizophrenia, temporal lobe epilepsy, and Alzheimer’s disease/mild cognitive impairment: a review	NEURORADIOLOGY	2.804	48	2	3.69
38	CA1 hippocampal neuronal loss in familial Alzheimer’s disease – Presenilin-1 E280A mutation is related to epilepsy	EPILEPSIA	5.886	48	3	2.53
39	Bexarotene reduces network excitability in models of Alzheimer’s disease and epilepsy	NEUROBIOLOGY OF AGING	4.673	46	4	5.11
40	Seizure resistance without parkinsonism in aged mice after tau reduction	NEUROBIOLOGY OF AGING	4.673	45	5	5
41	Early-Onset Network Hyperexcitability in Presymptomatic Alzheimer’s Disease Transgenic Mice Is Suppressed by Passive Immunization with Anti-Human APP/A beta Antibody and by mGluR5 Blockade	FRONTIERS IN AGING NEUROSCIENCE	5.75	43	11	7.17
42	Epigenetic suppression of hippocampal calbindin-D28k by Delta FosB drives seizure-related cognitive deficits	NATURE MEDICINE	53.44	42	17	7
43	Seizures in dominantly inherited Alzheimer disease	NEUROLOGY	9.91	42	9	6
44	Seizures in Alzheimer’s disease: a retrospective study of a cohort of outpatients	EPILEPTIC DISORDERS	1.819	41	7	3.15
45	Epilepsy presenting as AD: Neuroimaging, electroclinical features, and response to treatment	NEUROLOGY	9.91	41	0	1.95
46	Ectopic white matter neurons, a developmental abnormality that may be caused by the PSEN1 S169L mutation in a case of familial AD with myoclonus and seizures	JOURNAL OF NEUROPATHOLOGY AND EXPERIMENTAL NEUROLOGY	3.685	41	0	1.86
47	Alzheimer’s disease and late-onset epilepsy of unknown origin: two faces of beta amyloid pathology	NEUROBIOLOGY OF AGING	4.673	40	16	10
48	From here to epilepsy: the risk of seizure in patients with Alzheimer’s disease	EPILEPTIC DISORDERS	1.819	40	6	5.71
49	Alzheimer’s disease underlies some cases of complex partial status epilepticus - Clinical, radiologic, EEG, and pathologic correlations	JOURNAL OF CLINICAL NEUROPHYSIOLOGY	2.177	40	2	1.74
50	Early Onset of Hypersynchronous Network Activity and Expression of a Marker of Chronic Seizures in the Tg2576 Mouse Model of Alzheimer’s Disease	PLOS ONE	3.24	39	10	4.88
51	Chronic Temporal Lobe Epilepsy Is Associated with Enhanced Alzheimer-Like Neuropathology in 36 x Tg-AD Mice	PLOS ONE	3.24	39	6	3.55
52	Overview of cannabidiol (CBD) and its analogs: Structures, biological activities, and neuroprotective mechanisms in epilepsy and Alzheimer’s disease	EUROPEAN JOURNAL OF MEDICINAL CHEMISTRY	6.514	38	24	12.67
53	Adult-Onset Epilepsy in Presymptomatic Alzheimer’s Disease: A Retrospective Study	JOURNAL OF ALZHEIMER’S DISEASE	4.472	38	9	6.33
54	Seizures and dementia in the elderly: Nationwide Inpatient Sample 1999–2008	EPILEPSY AND BEHAVIOR	2.937	38	4	4.22
55	Senile myoclonic epilepsy: Delineation of a common condition associated with Alzheimer’s disease in Down syndrome	SEIZURE-EUROPEAN JOURNAL OF EPILEPSY	3.184	38	2	2.92
56	Presenilin-1 mutation Alzheimer’s disease: A genetic epilepsy syndrome?	EPILEPSY AND BEHAVIOR	2.937	37	4	3.08
57	Sleep EEG Detects Epileptiform Activity in Alzheimer’s Disease with High Sensitivity	JOURNAL OF ALZHEIMER’S DISEASE	4.472	36	9	6
58	Incidence and risk of seizures in Alzheimer’s disease: A nationwide population-based cohort study	EPILEPSY RESEARCH	3.045	33	11	4.13
59	Low brain ascorbic acid increases susceptibility to seizures in mouse models of decreased brain ascorbic acid transport and Alzheimer’s disease	EPILEPSY RESEARCH	3.045	33	2	4.13
60	Alzheimer’s Disease and Down Syndrome Rodent Models Exhibit Audiogenic Seizures	JOURNAL OF ALZHEIMER’S DISEASE	4.472	33	3	2.54
61	Paroxysmal slow cortical activity in Alzheimer’s disease and epilepsy is associated with blood-brain barrier dysfunction	SCIENCE TRANSLATIONAL MEDICINE	17.992	31	14	7.75
62	Prevalence, Semiology, and Risk Factors of Epilepsy in Alzheimer’s Disease: An Ambulatory EEG Study	JOURNAL OF ALZHEIMER’S DISEASE	4.472	30	12	6
63	Incidence of stroke and seizure in Alzheimer’s disease dementia	AGE AND AGEING	10.668	30	7	3.75
64	A presenilin 1 mutation (L420R) in a family with early onset Alzheimer disease, seizures and cotton wool plaques, but not spastic paraparesis	NEUROPATHOLOGY	1.906	30	1	1.88
65	Association of epileptiform abnormalities and seizures in Alzheimer disease	NEUROLOGY	9.91	29	16	9.67
66	Alzheimer-like amyloid and tau alterations associated with cognitive deficit in temporal lobe epilepsy	BRAIN	13.501	28	15	9.33
67	Tau-Induced Pathology in Epilepsy and Dementia: Notions from Patients and Animal Models	INTERNATIONAL JOURNAL OF MOLECULAR SCIENCES	5.924	28	10	5.6
68	A mouse model of Alzheimer’s disease displays increased susceptibility to kindling and seizure-associated death	EPILEPSIA	5.886	28	3	3.5
69	Early Seizure Activity Accelerates Depletion of Hippocampal Neural Stem Cells and Impairs Spatial Discrimination in an Alzheimer’s Disease Model	CELL REPORTS	9.423	27	14	6.75
70	Traumatic Brain Injury Increases the Expression of Nos1, A beta Clearance, and Epileptogenesis in APP/PS1 Mouse Model of Alzheimer’s Disease	MOLECULAR NEUROBIOLOGY	5.59	27	9	3.86
71	Alternative ion channel splicing in mesial temporal lobe epilepsy and Alzheimer’s disease	GENOME BIOLOGY	2.763	27	2	1.69
72	Hyperpolarization-activated cyclic nucleotide gated channels: a potential molecular link between epileptic seizures and A beta generation in Alzheimer’s disease	MOLECULAR NEURODEGENERATION	14.195	26	4	2.36
73	Untangling Alzheimer’s Disease and Epilepsy	EPILEPSY CURRENTS	7.5	26	2	2.36
74	Increased Epileptiform EEG Activity and Decreased Seizure Threshold in Arctic APP Transgenic Mouse Model of Alzheimer’s Disease	CURRENT ALZHEIMER’S RESEARCH	3.498	25	7	3.57
75	GSK3 beta and Tau Protein in Alzheimer’s Disease and Epilepsy	FRONTIERS IN CELLULAR NEUROSCIENCE	5.505	24	12	8
76	Alterations of Coherent Theta and Gamma Network Oscillations as an Early Biomarker of Temporal Lobe Epilepsy and Alzheimer’s Disease	FRONTIERS IN INTEGRATIVE NEUROSCIENCE	2.763	24	11	4.8
77	Seizure susceptibility in the APP/PS1 mouse’ model of Alzheimer’s disease and relationship with amyloid beta plaques	BRAIN RESEARCH	3.252	24	9	4
78	A possible significant role of zinc and GPR39 zinc sensing receptor in Alzheimer disease and epilepsy	BIOMEDICINE AND PHARMACOTHERAPY	6.53	24	2	3.43
79	Early onset familial Alzheimer disease with spastic paraparesis, dysarthria, and seizures and N135S mutation in PSEN1	ALZHEIMER DISEASE AND ASSOCIATED DISORDERS	2.703	22	5	1.47
80	Epilepsy and antiepileptic drug use in elderly people as risk factors for dementia	JOURNAL OF THE NEUROLOGICAL SCIENCES	3.181	22	4	1.38
81	Do we know how to diagnose epilepsy early in Alzheimer’s disease?	REVUE NEUROLOGIQUE	2.607	21	3	3.5
82	Increased risk of epilepsy in patients registered in the Swedish Dementia Registry	EUROPEAN JOURNAL OF NEUROLOGY	6.089	20	8	5
83	Seizures as an early symptom of autosomal dominant Alzheimer’s disease	NEUROBIOLOGY OF AGING	4.673	20	7	5
84	Inflammasome-derived cytokine IL18 suppresses amyloid-induced seizures in Alzheimer-prone mice	PROCEEDINGS OF THE NATIONAL ACADEMY OF SCIENCES OF THE UNITED STATES OF AMERICA	11.205	20	7	4
85	Epileptic Amnesic Syndrome revealing Alzheimer’s disease	EPILEPSY RESEARCH	3.045	20	0	1.82
86	*In Silico* Analyses for Key Genes and Molecular Genetic Mechanism in Epilepsy and Alzheimer’s Disease	CNS AND NEUROLOGICAL DISORDERS-DRUG TARGETS	4.388	19	4	3.8
87	Seizures Can Precede Cognitive Symptoms in Late-Onset Alzheimer’s Disease	JOURNAL OF ALZHEIMER’S DISEASE	4.472	19	0	1.58
88	Dementia, delusions and seizures: storage disease or genetic AD?	EUROPEAN JOURNAL OF NEUROLOGY	6.089	19	1	1.19
89	A Case Series of Epilepsy-derived Memory Impairment Resembling Alzheimer Disease	ALZHEIMER DISEASE AND ASSOCIATED DISORDERS	2.703	18	2	1.29
90	A novel mutation (L250V) in the presenilin 1 gene in a Japanese familial Alzheimer’s disease with myoclonus and generalized convulsion	JOURNAL OF THE NEUROLOGICAL SCIENCES	3.181	18	0	0.9
91	Epilepsy and Alzheimer’s Disease: Potential mechanisms for an association	BRAIN RESEARCH BULLETIN	4.079	17	7	5.67
92	Subclinical epileptiform activity during sleep in Alzheimer’s disease and mild cognitive impairment	CLINICAL NEUROPHYSIOLOGY	3.708	17	7	5.67
93	A Longitudinal Study of Epileptic Seizures in Alzheimer’s Disease	FRONTIERS IN NEUROLOGY	4.003	17	7	4.25
94	Seizures in Alzheimer’s disease are highly recurrent and associated with a poor disease course	JOURNAL OF NEUROLOGY	4.849	16	9	5.33
95	Pharmacotherapeutic strategies for treating epilepsy in patients with Alzheimer’s disease	EXPERT OPINION ON PHARMACOTHERAPY	3.889	16	5	3.2
96	From Molecular Circuit Dysfunction to Disease: Case Studies in Epilepsy, Traumatic Brain Injury, and Alzheimer’s Disease	NEUROSCIENTIST	7.519	16	1	2.29
97	Alzheimer beta-amyloid blocks epileptiform activity in hippocampal neurons	MOLECULAR AND CELLULAR NEUROSCIENCE	4.314	16	0	1.14
98	Memory disturbances and temporal lobe epilepsy simulating Alzheimer’s disease: a case report	FUNCTIONAL NEUROLOGY	1.855	16	0	0.8
99	Epileptic seizures in autosomal dominant forms of Alzheimer’s disease	SEIZURE-EUROPEAN JOURNAL OF EPILEPSY	3.184	15	3	3
100	DBA/2J Genetic Background Exacerbates Spontaneous Lethal Seizures but Lessens Amyloid Deposition in a Mouse Model of Alzheimer’s Disease	PLOS ONE	4.24	15	0	1.88

As shown in Table 1, The 100 most-cited articles received a total of 6743 citations (according to Web of science, WOS). The median number citations was 39, with a range of 15–433. For annual citations, the mean value was 7.41 with a range of 0.8–35.70. Seventeen papers were cited more than 100 times, and 36 were cited more than 50 times. The review entitled “Epilepsy and Cognitive Impairments in Alzheimer Disease” from Palop and Mucke (2009) was the most-cited publication (*n* = 433).

### Publication years

**FIGURE 2:**
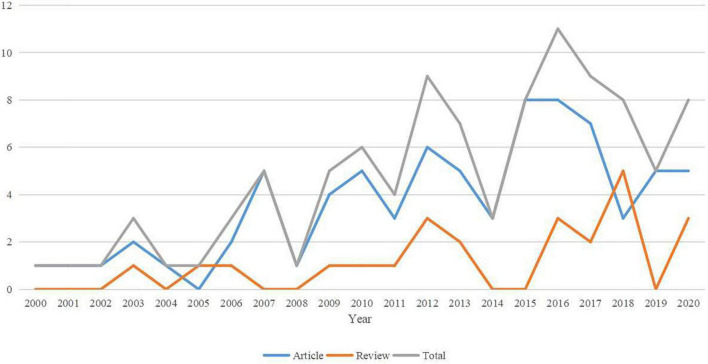
The number of publications among different types of papers according to publication year.

As shown in Figure 2, the top 100 most-cited papers were published from 2000 to 2020. Overall, publications showed a fluctuating upward trend. The largest number of studies was published in 2016 (*n* = 11), including eight articles and three reviews. The number of papers published in 2011 was higher than the number of papers published between 2000 and 2010.

### Contributions of countries

**FIGURE 3:**
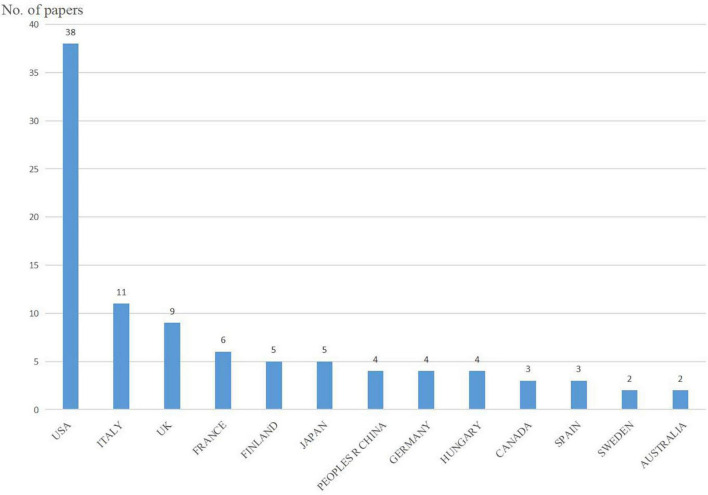
Countries among the top 100 most-cited papers.

Overall, 22 countries contributed to the included studies, with nine countries publishing only one study. The United States was the largest contributor of studies (38%), followed by Italy (11%) and the United Kingdom (9%). The contributing countries are shown in Figure 3.

**FIGURE 4:**
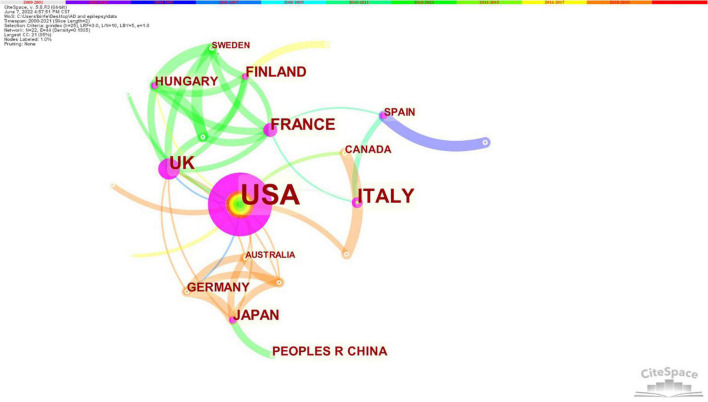
Contributions of countries. Purple rings indicated that these countries/regions had greater centrality (no less than 0.1). The size of the circle represents the number of papers published by the country. The shorter the distance between two circles, the greater the cooperation between the two countries. United States was the most influential research country.

Generating a country map using CiteSpace resulted in 22 nodes and 44 links (Figure 4). The 100 papers were published by research groups in 22 countries. The top five countries were the United States, Italy, the United Kingdom, France, and Finland. The top three countries in terms of centrality were the United States (0.93), the United Kingdom (0.44), and France (0.21). An analysis in terms of publication and centrality indicated that the United States, the United Kingdom, Italy, and France were the main research powers in this research. United States has established cooperation with 12 countries, and the strongest collaborations were identified between United States, Canada, Israel, Greece, India, and Switzerland.

### Contributions of institutions

A total of 178 institutions published at least one top-cited paper, the distribution of institutions was very scattered, with 21 (11.80%) institutions publishing two papers and 148 (83.15%) institutions publishing only one paper. A small number of institutions accounted for a high proportion of the highest cited papers. Table 2 shows that the top nine institutions collectively published at least three papers. Baylor College of Medicine from United States topped the list with eight papers.

**TABLE 2:** Top nine institutions with at least three papers in the top 100 most-cited papers.

Rank	Institution	No. of papers	Centrality
1	Baylor College of Medicine	8	0.04
2	University of Eastern Finland	5	0
3	Columbia University	4	0.06
4	Johns Hopkins University	4	0.04
5	Kuopio University Hospital	3	0
6	Semmelweis University	3	0
7	National Institute of Clinical Neurosciences	3	0
8	New York University	3	0.01
9	Centre National de la Recherche Scientifique	3	0

**FIGURE 5:**
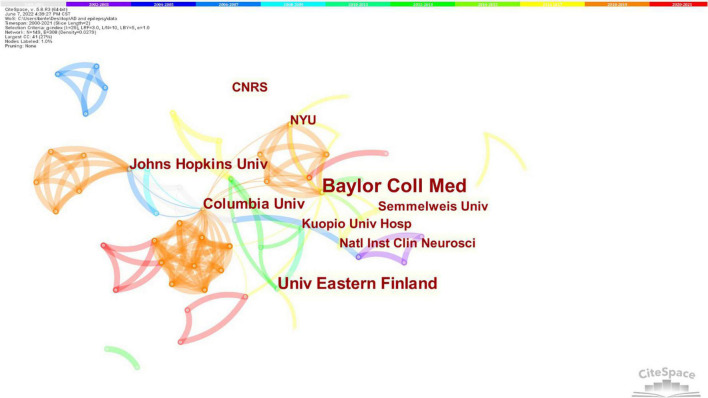
Contributions of institutions. Nodes represent institutes, and the size of each node corresponds to the co-occurrence frequency of the institutes. The size of the circle represents the number of papers published by the institute. The shorter the distance between two circles, the greater the cooperation between the two institutes. Columbia University was the most influential research institutions.

Figure 5 shows that generating an institution map resulted in 149 nodes and 308 links. The top 100 publications were distributed among 149 research institutions. The top five institutions were Baylor College Medicine, University of Eastern Finland, Columbia University, and Johns Hopkins University and Kuopio University Hospital. The top four institutions in terms of centrality were Columbia University (0.06), Baylor College of Medicine (0.04), Johns Hopkins University (0.04) and Indiana University (0.03). Analysis in terms of centrality indicated that Columbia University was the most influential research institution.

### Distribution of journals

The 100 most-cited articles were published in 59 journals; 18 journals published more than one study, which were distributed in the four partitions of Journal Citation Reports (JCR) (McVeigh, 2008). The JCR data provide both detailed journal information and flexible context, and category information to allow people to understand the way each journal functions in the literature. The major contributing journals are presented in Table 3. The top five journals that published the 100 most-cited AD and epilepsy studies included Journal of Alzheimer’s Disease (*n* = 8), Epilepsia (*n* = 6), Epilepsy and Behavior (*n* = 4), Neurobiology of Aging (*n* = 4) and Epilepsy Research (*n* = 4). With regard to the average citation number per paper, Journal of Neuroscience ranked first with a mean of 20.98 citations per paper, followed by Nature Medicine, with a mean of 17.84.

**TABLE 3:** Journals contributed ≥ 2 papers in the top 100 most-cited papers.

Journal	JCR category	Frequency	JCR partition	IF (2020)	IF (5 year)	Citations WOS Mean ± SD	Citations in 2021 Mean ± SD	Citations per year Mean ± SD
JOURNAL OF ALZHEIMER’S DISEASE	Neurosciences	8	Q2	4.472	4.851	40.50 ± 14.21	9.5 ± 6.66	5.65 ± 2.74
EPILEPSIA	Clinical Neurology	6	Q1	5.886	7.1211	130 ± 136.84	12.5 ± 13.26	9.51 ± 7.82
EPILEPSY AND BEHAVIOR	Behavioral Sciences; Clinical Neurology; Psychiatry	4	Q2, Q3, Q3	2.937	3.224	67 ± 36.63	10 ± 8.98	6.0 ± 2.76
EPILEPSY RESEARCH	Clinical Neurology	4	Q3	3.045	3.143	37.75 ± 19.17	5.5 ± 5.32	3.88 ± 1.50
NEUROBIOLOGY OF AGING	Geriatrics and Gerontology; Neurosciences	4	Q2, Q2	4.673	5.164	37.75 ± 12.12	8 ± 5.48	6.28 ± 2.48
ALZHEIMER DISEASE AND ASSOCIATED DISORDERS	Clinical Neurology; Pathology	3	Q3, Q3	2.703	3.007	33 ± 22.61	6 ± 4.58	3.73 ± 4.07
ARCHIVES OF NEUROLOGY	Clinical Neurology	3	Q1	7.419	7.249	234 ± 180.16	23.67 ± 14.19	17.27 ± 12.25
BRAIN	Clinical Neurology; Neurosciences	3	Q1, Q1	13.501	14.25	81.33 ± 48.56	24.67 ± 8.51	15.17 ± 5.08
EUROPEAN JOURNAL OF NEUROLOGY	Clinical Neurology; Neurosciences	3	Q1, Q1	6.089	5.308	32.33 ± 22.23	5.67 ± 4.04	3.27 ± 1.93
JOURNAL OF NEUROSCIENCE	Neurosciences	3	Q1	6.167	6.993	248.33 ± 137.45	37 ± 14.93	20.98 ± 7.23
NEUROLOGY	Clinical Neurology	3	Q1	9.91	10.664	37.33 ± 12.12	8.33 ± 8.02	5.87 ± 3.86
PLOS ONE	Multidisciplinary Sciences	3	Q3	3.24	3.788	31 ± 13.86	5.33 ± 5.03	3.44 ± 1.50
EPILEPSY CURRENTS	Clinical Neurology	2	Q1	7.5	8.772	48.50 ± 31.82	6.50 ± 6.36	4.41 ± 2.89
EPILEPTIC DISORDERS	Clinical Neurology	2	Q4	1.819	2.352	40.50 ± 0.71	6.50 ± 0.71	4.43 ± 1.81
JOURNAL OF NEUROCHEMISTRY	Biochemistry and Molecular Biology; Neurosciences	2	Q2, Q1	5.372	5.69	80.50 ± 34.65	7 ± 2.83	5.89 ± 0.41
JOURNAL OF THE NEUROLOGICAL SCIENCES	Clinical Neurology; Neurosciences	2	Q3, Q3	3.181	3.403	20 ± 2.83	2 ± 2.83	1.14 ± 0.34
NATURE MEDICINE	Biochemistry and Molecular Biology; Cell Biology; Research and Experimental Medicine	2	Q1, Q1, Q1	53.44	49.248	107.00 ± 91.92	35.50 ± 26.16	17.84 ± 15.32
SEIZURE-EUROPEAN JOURNAL OF EPILEPSY	Clinical Neurology; Neurosciences	2	Q3, Q3	3.184	3.729	26.50 ± 16.26	2.50 ± 0.71	2.96 ± 0.06

**FIGURE 6:**
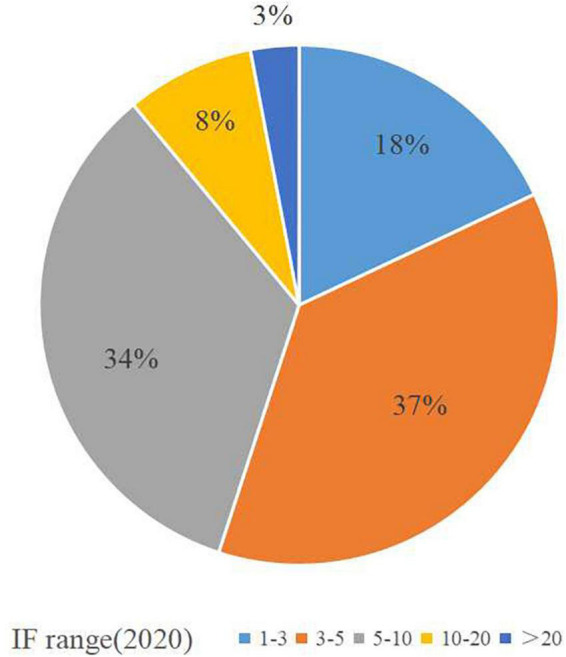
The number of articles corresponding to different IF in the top 100 most-cited papers.

The IF for journals in the top 100 most-cited papers ranged from 1.819 to 53.44, among which 37 journals had an IF between 3 and 5, 34 journals had an IF between 5 and 10, and 3 journals had an IF above 20 (Figure 6).

In Table 4, the top 100 papers were classified into different study fields on the basis of WOS categories. The leading WOS category was “Clinical Neurology” (*n* = 54), following by “Neurosciences” (*n* = 49) and “Behavioral Sciences” (*n* = 10).

**TABLE 4:** The number of study fields on the basis of WOS categories.

WoS Categories	Frequency
Clinical Neurology	54
Neurosciences	49
Behavioral Sciences	10
Geriatrics and Gerontology	8
Multidisciplinary Sciences	5
Psychiatry	5
Pathology	5
Biochemistry and Molecular Biology	5

### Major contributing authors

Overall, a total of 606 authors contributed to the 100 studies. There was wide, disparate authorship of first authors, with 90 different first authors represented in the 100 included publications. A total of four contributors published the most articles, namely Tanila Heikki, Noebels Jeffrey, Pitkänen, Asla and Scharfman, Helen E, who all published five publications, the total number of citations for the papers were 629, 592, 578, and 234 respectively. Only three authors have published three studies as a first author. Keith A. Vossel from the University of California, as a first author and corresponding author, had the largest number of total citations in 2021 (*n* = 185). Table 5 presents results for authors who contributed three or more of the 100 most-cited papers.

**TABLE 5:** Major contributing authors in the top 100 most-cited list.

Author	Frequency	Total citations WOS	Total citation in 2021	First author	Correspond author	Co-author	Constitution	Country
Tanila, Heikki	5	629	92	0	2	4	Kuopio University Hospital	Finland
Noebels, Jeffrey	5	592	110	1	2	3	Baylor Coll Medicine	United States
Pitkänen, Asla	5	578	84	0	2	3	Kuopio University Hospital	Finland
Scharfman, Helen E.	5	234	33	1	2	3	New York University Langone Medical Center	United States
Vossel, Keith A.	4	830	185	3	4	0	University of California	United States
Beagle, Alexander J.	3	617	121	1	0	2	University of California	United States
Miller, Bruce L.	3	701	163	0	0	3	University of California	United States
Mucke, Lennart	3	991	138	0	0	3	University of California	United States
Scarmeas, Nikolaos	3	393	55	1	2	1	University of California	United States
Szûcs, Anna	3	128	31	0	0	3	Semmelweis University	Hungary
Barcs, Gábor	3	128	31	0	0	3	Semmelweis University	Hungary
Horváth, András	3	128	31	3	3	0	Semmelweis University	Hungary
Kamondi, Anita	3	128	31	0	0	3	Semmelweis University	Hungary
Blanc, Frédéric	3	120	22	0	0	3	University of Strasbourg	France
Cretin, Benjamin	3	94	18	3	3	0	University of Strasbourg	France
Sellal, Francois	3	120	22	0	0	3	University of Strasbourg	France
Gurevicius, Kestutis	3	172	22	1	1	2	Kuopio University Hospital	Finland
Ziyatdinova, Sofya	3	146	21	2	1	1	University of Eastern Finland	Finland
Larner, A. J.	3	214	15	2	3	0	Walton Centre for Neurology and Neurosurgery	United Kingdom

### Analysis of co-occurring keywords

**FIGURE 7:**
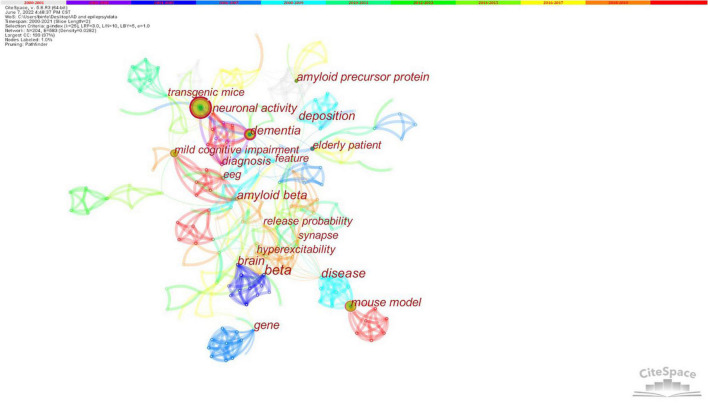
Analysis of co-occurrence Keywords. Nodes represent keywords, and the size of each node corresponds to the co-occurring frequency of the keywords. The color of the lines that appear together between keywords indicates chronological order: grey represents the oldest, and orange the newest. “beta” had a highest degree of centrality.

CiteSpace was used to extract the keywords from the top 100 papers on AD and epilepsy. A network analysis of the author’s keywords or subject words was carried out during the publication time of the articles, namely, 2000–2021. Table 6 shows that the top five keywords are epilepsy (*n* = 41), dementia (*n* = 21), mouse model (*n* = 21), mild cognitive impairment (*n* = 13), and Alzheimer’s disease (*n* = 11). The greater the centrality value, the more cooperation between the node and other nodes. Figure 7 shows that “beta” (centrality: 0.39), “amyloid beta” (centrality: 0.29), “hyperexcitability” (centrality: 0.29) and “disease” (centrality: 0.29) had a high degree of centrality during this period. A comprehensive analysis of centrality showed that “beta” (*n* = 7, centrality: 0.39), “disease” (*n* = 3, centrality: 0.29), “dementia” (*n* = 21, centrality: 0.25) and “brain” (*n* = 4, centrality: 0.25) are the most influential keywords in this field.

**TABLE 6:** Frequency of co-occurring keywords.

Keyword	Frequency	Centrality
Epilepsy	41	0.13
Dementia	21	0.25
Mouse model	21	0.11
Mild cognitive impairment	13	0.14
Alzheimer’s disease	11	0.17
Amyloid precursor protein	10	0.13
Diagnosis	8	0.23
Elderly patient	8	0.2
Neuronal activity	7	0.23
Beta	7	0.39
EEG	6	0.09
Transgenic mice	6	0.12
Amyloid beta	6	0.29

## Discussion

The aim of this paper was to perform a bibliometric study of the top 100 most-cited publications on AD and epilepsy over the last 22 years. To the best of our knowledge, this was the first study to provide an overview the current main status of development, hot spots of study, and the future trends in AD and epilepsy. Our study is of great significance for students, researchers, and clinicians working in the field. Here, we summarize several characteristics of these papers to help understand the history and professional prospects comprehensively.

The average number of citations for the 100 most-cited clinical trials in AD and epilepsy was 67.43 (range: 15–433), well below the number of citations observed in general neuroscience articles (3,087) (Yeung et al., 2017). However, citation counts are not a direct measure of scientific quality and importance. The number of citations can be regarded as a relatively reasonable index to evaluate the quality of papers, which varies in different sub disciplines and depends on the size of the scientific community. Citation count analysis might be related to journal IF, publication frequency, and publication year. In general, an article with 100 or more citations is considered a “classic” in the research field and might even be a seminal paper (Xiong et al., 2021). However, our study showed that only 17 papers were cited more than 100 times, this can possibly come from the fact that comorbid epilepsy in AD may not be an interesting topic for many clinicians. In fact, cognitivists dealing with AD patients are not highly aware of epilepsy and, inversely, epileptologists may be poorly informed of dementing diseases. Therefore, the AD-epilepsy topic may have interest only for a small research community, which can result in a relatively low citation index.

The most-cited publication was “Epilepsy and Cognitive Impairments in Alzheimer’s Disease” (Palop and Mucke, 2009), wrote by Palop and Mucke (2009), with 433 total citations, 31 annual citations, and 39 citations in 2021. It may be relevant to current research and have far-reaching implications. However, in general, consensus and position papers, guides, and systematic reviews receive more citations than original articles, which is a bias that must be considered in citation analysis. The article that ranked second, which was based on a “Amyloid beta-Induced Neuronal Hyperexcitability Triggers Progressive Epilepsy” (Minkeviciene et al., 2009) had a total of 405 citations, 28.93 annual citations and 54 citations in 2021; it was published in Journal of Neuroscience in 2009 and written by Minkeviciene et al. (2009). The team performed video-EEG recordings in mice carrying mutant human APPswe and PS1dE9 genes (APdE9 mice) and their wild-type littermates, identifying fibrillar Abeta may be the cause of epileptiform activity.

The number of papers published has increased in recent years. Although the most cited publications tend to be from the first few years (2000–2010), the number of highly cited articles published from 2011–2020 is nearly three times that published from 2000–2010. In 2016, the number of publications was the largest, reaching 11, which showed that a recently published article is likely to gradually improve the quality of research in recent years and have a potential academic importance in the future.

The majority of research originated from the United States (38%). A bibliometric analysis of the most cited articles in neurocritical care research showed that United States was the country with most articles (60, 35 primary research) and citations (6115) among the top 100 (Ramos et al., 2019). Baylor College of Medicine from United States topped the list with eight papers. The distribution of country and institutions was very scattered. The United States and its institutions have played a leading role in AD and epilepsy research. The United States was also ranked first in other fields of neurology (Xiong et al., 2021), and it is also a research leader in terms of quality and quantity. However, compared with other studies, our study showed that the cooperation between countries and institutions was not close (Yin et al., 2019). In fact, just like the study of Parkinson’s disease (Li et al., 2008), more cooperation between different countries and institutions might be an effective way to promote the development of AD and epilepsy research worldwide. Contrastingly, distribution analysis showed that research was widespread all over the world and the diseases had research value.

Baylor College of Medicine and Columbia University played important roles in in the field of AD and epilepsy research. Baylor College of Medicine, as one of the top colleges and universities in Texas, United States, focused on cooperative research programs, discovered basic insights into human health and diseases through extensive interdisciplinary and interdisciplinary cooperation, and applied their findings to develop new diagnostic tools and treatments. Columbia University is a world-class private research university located in Manhattan, NY, United States. It carried out extensive research in the field of neuroscience.

The 100 included articles were published in 59 journals, among which 18 journals published more than one study. The papers were distributed in four partitions of JCR. The IF of the top five journals was less than six. The top journal among the list of the 100 most-cited studies was Journal of Alzheimer’s Disease (IF = 4.472). The leading WOS categories focused on “Clinical Neurology” and “Neurosciences,” with few interdisciplinary studies. Resulting from its interdisciplinary nature, Psycho-Oncology has been subject to extensive interdisciplinary research, which has provided great help for the development of the discipline (Fox et al., 2021). Therefore, we look forward to more interdisciplinary research.

Author analysis revealed a network of core author collaborations in the field of AD and epilepsy research. This information might be relevant to clinical researchers and research institutions who are searching for a network of research leaders in the field to explore potential collaborations. Our authorship analysis does not show as many authors as other areas in research. The most contributing author was Tanila, Heikki from Kuopio University Hospital from Finland. Related research focuses on the pathogenesis of epilepsy in mouse models of AD, which is a research hotspot in this field.

Keyword analysis showed that “beta,” “amyloid beta,” “hyperexcitability,” “disease,” “dementia,” and “brain” had high influential. indicating that these research directions are very significant. This result showed that researchers have begun to extend their research to the pathogenesis. These results also show that AD and epilepsy is still a disease that requires to be solved urgently. We must explore the deficiencies and innovations in this field, such as treatment, pathological mechanism, and disease management on improve the quality of life of patients.

### Limitations

The present study has some limitations. First, there are several intrinsic limitations of using citation analysis to evaluate the academic importance of a specific article, author, or journal. There is a certain bias in citation analysis, such as the fact that papers written in English, papers that can be accessed through open access, and papers published in journals with high IFs are more likely to be cited. In addition, through a “snowball effect,” people tended to cite publications that are already highly cited (Yeung et al., 2017). We selected the top 100 papers, but citation searches are “time-dependent,” older articles are likely to be cited more often, and the newest list of highly cited articles may be dominated by some older articles. Furthermore, citation analysis might severely underestimate the impact of clinical research as compared to basic research (van Eck et al., 2013). Second, the search was limited to the WOS database. It did not record citations by textbooks and other databases. Our study only selected papers written in English, which might have yielded an incomplete search.

## Conclusion

We identified the 100 most cited papers in the field of AD and epilepsy. By reviewing these top cited papers, researchers can immediately understand the hot topics and research collaborations on AD and epilepsy, and improve their work. This study shows that the relationship, mechanism, and treatment of AD and epilepsy have been widely studied, and in recent years, this field has shown new vitality; however, there is a general lack of cooperation between countries, and the mechanism of epilepsy and AD is unclear, which deserves further study.

## Data availability statement

The datasets presented in this study can be found in online repositories. The names of the repository/repositories and accession number(s) can be found in the article/supplementary material.

## Author contributions

G-FZ and YG designed the study. G-FZ drafted and edited the manuscript. G-FZ and W-XG analyzed the data. G-FZ, Z-Y-RX, YG, and W-XG revised the manuscript. All authors contributed to the article and approved the submitted version.

## Conflict of interest

The authors declare that the research was conducted in the absence of any commercial or financial relationships that could be construed as a potential conflict of interest.

## Publisher’s note

All claims expressed in this article are solely those of the authors and do not necessarily represent those of their affiliated organizations, or those of the publisher, the editors and the reviewers. Any product that may be evaluated in this article, or claim that may be made by its manufacturer, is not guaranteed or endorsed by the publisher.

## References

[B1] Beghi E. (2020). The Epidemiology of Epilepsy. *Neuroepidemiology* 54 185–191. 10.1159/000503831 31852003

[B2] Cretin B. (2021). Treatment of Seizures in Older Patients with Dementia. *Drugs Aging* 38 181–192. 10.1007/s40266-020-00826-2 33314010

[B3] Fox S. Lynch J. D’Alton P. Carr A. (2021). Psycho-Oncology: a Bibliometric Review of the 100 Most-Cited Articles. *Healthcare* 9:1008. 10.3390/healthcare9081008 34442145 PMC8393329

[B4] Guo Y. Xu Z. Y. R. Cai M. T. Gong W. X. Shen C. H. (2021). Epilepsy With Suicide: a Bibliometrics Study and Visualization Analysis via CiteSpace. *Front. Neurol.* 12:823474. 10.3389/fneur.2021.823474 35111131 PMC8802777

[B5] Lehmann L. Lo A. Knox K. M. Barker-Haliski M. (2021). Alzheimer’s Disease and Epilepsy: a Perspective on the Opportunities for Overlapping Therapeutic Innovation. *Neurochem. Res.* 46 1895–1912. 10.1007/s11064-021-03332-y 33929683 PMC8254705

[B6] Li T. Ho Y. S. Li C. Y. (2008). Bibliometric analysis on global Parkinson’s disease research trends during 1991-2006. *Neurosci. Lett.* 441 248–252. 10.1016/j.neulet.2008.06.044 18582532

[B7] Liu J. Wang L. N. (2021). Treatment of epilepsy for people with Alzheimer’s disease. *Cochrane Database Syst. Rev.* 12:CD011922. 10.1002/14651858.CD011922.pub4 33973646 PMC8111487

[B8] McVeigh M. E. (2008). Neurology in the journal citation reports. *Neurology* 71 1848–1849. 10.1212/01.wnl.0000338902.81462.88 19047557

[B9] Minkeviciene R. Rheims S. Dobszay M. B. Zilberter M. Hartikainen J. Fülöp L. (2009). Amyloid beta-induced neuronal hyperexcitability triggers progressive epilepsy. *J. Neurosci.* 29 3453–3462. 10.1523/JNEUROSCI.5215-08.2009 19295151 PMC6665248

[B10] Niu H. Álvarez-Álvarez I. Guillén-Grima F. Aguinaga-Ontoso I. (2017). Prevalence and incidence of Alzheimer’s disease in Europe: a meta-analysis. *Neurologia* 32 523–532. 10.1016/j.nrl.2016.02.016 27130306

[B11] Palop J. J. Mucke L. (2009). Epilepsy and Cognitive Impairments in Alzheimer Disease. *Arch. Neurol.* 66 435–440. 10.1001/archneurol.2009.15 19204149 PMC2812914

[B12] Ramos M. B. Koterba E. Rosi Júnior J. Teixeira M. J. Figueiredo E. G. A. (2019). Bibliometric Analysis of the Most Cited Articles in Neurocritical Care Research. *Neurocrit. Care* 31 365–372. 10.1007/s12028-019-00731-6 31087256

[B13] Samson W. N. van Duijn C. M. Hop W. C. Hofman A. (1996). Clinical features and mortality in patients with early-onset Alzheimer’s disease. *Eur. Neurol.* 36 103–106. 10.1159/000117218 8654478

[B14] Sen A. Capelli V. Husain M. (2018). Cognition and dementia in older patients with epilepsy. *Brain* 141 1592–1608. 10.1093/brain/awy022 29506031 PMC5972564

[B15] Struble R. G. Ala T. Patrylo P. R. Brewer G. J. Yan X. X. (2010). Is brain amyloid production a cause or a result of dementia of the Alzheimer’s type? *J. Alzheimers Dis.* 22 393–399. 10.3233/JAD-2010-100846 20847431 PMC3079347

[B16] van Eck N. J. Waltman L. van Raan A. F. Klautz R. J. Peul W. C. (2013). Citation analysis may severely underestimate the impact of clinical research as compared to basic research. *PLoS One.* 8:e62395. 10.1371/journal.pone.0062395 23638064 PMC3634776

[B17] Wu M. Q. Wu D. Q. Hu C. P. Iao L. S. (2021). Studies on Children With Developmental Coordination Disorder in the Past 20 Years: a Bibliometric Analysis via CiteSpace. *Front Psychiatry* 12:776883. 10.3389/fpsyt.2021.776883 34938213 PMC8685384

[B18] Xiong H. Y. Liu H. Wang X. Q. (2021). Top 100 Most-Cited Papers in Neuropathic Pain From 2000 to 2020: a Bibliometric Study. *Front. Neurol.* 12:765193. 10.3389/fneur.2021.765193 34867750 PMC8632696

[B19] Yeung A. W. K. Goto T. K. Leung W. K. (2017). At the Leading Front of Neuroscience: a Bibliometric Study of the 100 Most-Cited Articles. *Front. Hum. Neurosci.* 11:363. 10.3389/fnhum.2017.00363 28785211 PMC5520389

[B20] Yin X. Cheng F. Wang X. Mu J. Ma C. Zhai C. (2019). Top 100 cited articles on rheumatoid arthritis: a bibliometric analysis. *Medicine* 98:e14523. 10.1097/md.0000000000014523 30813156 PMC6408095

